# Fine-scale nutrient and carbonate system dynamics around cold-water coral reefs in the northeast Atlantic

**DOI:** 10.1038/srep03671

**Published:** 2014-01-20

**Authors:** Helen S. Findlay, Sebastian J. Hennige, Laura C. Wicks, Juan Moreno Navas, E. Malcolm S. Woodward, J. Murray Roberts

**Affiliations:** 1 Plymouth Marine Laboratory, Prospect Place, West Hoe, Plymouth, England, PL1 3DH, UK; 2 Centre for Marine Biodiversity & Biotechnology, School of Life Sciences, Heriot-Watt University, Edinburgh, Scotland, EH14 4AS, UK; 3 Scottish Association for Marine Science, Oban, PA37 1QA, UK; 4 Center for Marine Science, University of North Carolina, Wilmington, 601 S. College Road, Wilmington, North Carolina, 28403-5928, USA

## Abstract

Ocean acidification has been suggested as a serious threat to the future existence of cold-water corals (CWC). However, there are few fine-scale temporal and spatial datasets of carbonate and nutrients conditions available for these reefs, which can provide a baseline definition of extant conditions. Here we provide observational data from four different sites in the northeast Atlantic that are known habitats for CWC. These habitats differ by depth and by the nature of the coral habitat. At depths where CWC are known to occur across these sites the dissolved inorganic carbon ranged from 2088 to 2186 μmol kg^−1^, alkalinity ranged from 2299 to 2346 μmol kg^−1^, and aragonite Ω ranged from 1.35 to 2.44. At two sites fine-scale hydrodynamics caused increased variability in the carbonate and nutrient conditions over daily time-scales. The observed high level of variability must be taken into account when assessing CWC sensitivities to future environmental change.

Specific dynamics such as temperature, salinity, oxygen availability, light, and water currents, have been shown to control reef and coral growth on tropical shallow-water zooxanthellate coral reefs [e.g.^1^,^2^], but also, more recently, on cold-water non-zooxanthellate coral reefs [e.g.^3^,^4^]. While the physical dynamics are clearly important, the biogeochemical dynamics (interlinked with the physics), such as the carbonate ion concentration or nutrients^5^, are also recognized to contribute to reef and coral growth. On tropical coral reefs, carbon and nutrient dynamics have been shown to be highly variably^6^,^7^,^8^, but the presence of the reefs themselves can also contribute to the variability in carbon and nutrients over daily and seasonal time-scales^9^,^10^,^11^.

Dullo *et al.*^4^ suggested that cold-water coral (CWC) reefs are not randomly distributed but instead can be found at certain density ranges, specifically from 27.35 to 27.65. This density pre-requisite has been confirmed in more recent observations of both deep and shallow CWC reefs [e.g.^12^,^13^], however not all CWC habitats are found in such a narrow density range^14^, and it is highly relevant we consider all these types of habitats. While this identifier has been used to locate living CWC reefs, Dullo *et al.*^4^ also highlight the need for further research on related processes including nutrient inventories, carbonate chemistry and, critically, mechanisms by which food is supplied to the benthos. At present, nutrient and carbonate system data are primarily only available from global studies such as the World Ocean Atlas (WOA)^15^ and the GLobal Ocean Data Analysis Project (GLODAP)^16^, and while useful for global assessments, provide very limited fine-scale spatial and temporal coverage that would describe a detailed baseline of the physicochemical environment that these CWC reef organisms experience. *McGrath et al.*^17^ analyzed data from local and World Ocean Circulation Experiment (WOCE) cruises for the Rockall Trough between 1991–2010, providing a longer-term temporal analysis of the carbonate system at a more local scale, however the main focus was on the water column structure and change through time, as opposed to the CWC reef environmental conditions. The WOA and GLODAP datasets have been used to provide broad environmental envelopes for conditions that CWCs could exist on a global scale [e.g.^4^], but they are more limited in their use for regional analysis. Lunden *et al.*^18^ provided a comprehensive survey of carbonate chemistry measurements specifically surrounding CWC reefs in the Gulf of Mexico, and Flögel *et al.*^19^ more recently provided a summary of carbonate chemistry measurements for CWC in the wider North Atlantic region. However, in general fine-scale data is still hard to come by for these important organisms and their habitats, yet these CWC exist in highly dynamic environments, and their local distribution may be associated with this natural variability. In the present study, we aim to assess the variability of nutrient and carbonate system dynamics across a range of temporal and spatial scales at four sites of known (but different) CWC reef habitats in the northeast Atlantic, representing a large diversity of CWC habitats surveyed in one research expedition.

The Rockall Bank is situated in the northeast Atlantic, approximately 400 km west of the Outer Hebrides (Fig. 1a). The depth ranges from over 1000 m at the base of the Bank, to 200 m across much of the top of the bank. Oceanic banks such as the Rockall Bank are characteristic in deviating major ocean currents to run along their flanks^20^, and these hydrodynamic regimes facilitate colonisation of CWC reefs^21^,^22^. The flanks of the Rockall Bank are known to contain extensive CWC reef habitats^22^,^23^. Two such sites are the Logachev coral carbonate mounds on the southwest flank^24^; and a site on the northwest flank (Fig. 1a, b), the Pisces site, first examined by Wilson^21^ using the *Pisces III* submersible in 1973 (Fig. 1a), which includes a benthic ecosystem incorporating ‘Wilson ring’ patches of *Lophelia pertusa* reef habitat (Fig. 1c). Coral carbonate mounds, such as those at the Logachev site (Fig. 1d), are formed from successive periods of interglacial coral growth, whereas CWC patches (e.g. Pisces) and reefs (e.g. Mingulay Reef Complex) are ‘single generation’ Holocene accumulations^25^,^26^. Living reefs have been found at depths from 400 to 800 m at Logachev and from 200 to 400 m at Pisces. To contrast these deeper reefs, the third site is the Mingulay Reef Complex (MRC), a shallow CWC reef area consisting of a number of individual reefs (Fig. 1e), including Mingulay Area 01 (MA01) and Banana reef. The MRC is located on the European continental shelf, east of the Outer Hebrides, Scotland (Fig. 1a), where living reefs are found at depths from 120 to 190 m^27^,^28^. The final site was the Hebrides Terrace Seamount (HTS), which is found in approximately 2000 m water depth near the continental slope (Fig. 1a). Novel visual surveys of the seamount benthos have revealed previously unknown ‘coral garden’ habitats some of which are structured by the colonial scleractinian *Solenosmilia variabilis* (Fig. 1f).

In the present study, additional focused sampling was carried out over a tidal cycle at three stations (MA01, ‘Logachev South (LS)’ and ‘Logachev North (LN)’, Fig. 1) to investigate the influence of the fine-scale ocean hydrodynamics on the environmental conditions at the depth of the CWC reefs. At the MRC, there is a known tidally-driven downwelling, and the detailed dynamics of the biogeochemical conditions of that site are described in Findlay *et al.*^29^ therefore, only the relative variability is summarised here. In the region of the southwest Rockall Trough, containing the Logachev sites, there are known diurnal internal waves which cause vertical displacement of water masses resulting in relatively large fluctuations in temperature and salinity^30^. Carbonate and nutrient dynamics were therefore measured over a tidal period for both north (LN) and south (LS) of one of the coral carbonate mounds at Logachev.

## Results

### Temperature, salinity & water masses

The northeast Atlantic is a complex region with numerous water masses. The MRC showed distinctly different temperature and salinity properties from all the other sites, which was primarily due to the coastal influence, and addition of freshwater run-off (Fig. 2). All the other sites (Logachev, Pisces and HTS) surrounding the Rockall Bank and Trough area, showed relatively similar properties throughout the water column (Fig. 2). The surface water (SW, Fig. 2) was indicated by an increase in temperature, but with little change in salinity. Below the surface water layer was found predominantly East North Atlantic Water (ENAW, Fig. 2), at depths between about 200 m to 700 m. Only Logachev and HTS were deeper than 700 m. At Logachev, below 700 m, Wyville-Thomson Overflow Water (WTOW, Fig. 2) was identified^20^, and at HTS, the WTOW was present at depths down to around 1200 m. Below 1200 m the water was influenced by colder fresher Labrador Sea Water (LSW, Fig. 2). The water mass properties used here are taken from McGrath *et al.*^20^ specifically for the Rockall Trough region.

### Fluorescence (chlorophyll), oxygen & particle attenuation coefficient (*Cp*)

Chlorophyll fluorescence and *Cp* were correlated at all sites, such that as fluorescence increased, *Cp* increased (overall slope *β* = 0.67, *r* = 0.509, *p* < 0.0001, supplementary Fig. S1 online). The slope of the fluorescence-*Cp* relationship was significantly different for each site (MRC *β* = 0.644, Logachev *β* = 0.3724, HTS *β* = 0.8143 and Pisces *β* = 1.946; supplementary Fig. S2 online). At MA01 there was a second increase in *Cp* towards the reef, which did not have a concomitant increase in chlorophyll (labeled as ‘A’ in Supplementary Fig. S1 online). This increase at depth was also evident at Logachev and at the HTS, and is likely to be due to resuspension of particulate matter from the seabed. There was also a deviation from the general trend in the surface waters at Pisces and the HTS, where fluorescence decreased without *Cp* decreasing (labeled as ‘B’ in Supplementary Fig. S1 online). At these sites there was clear evidence of a coccolithophore bloom in the upper water column at this time (late May-Early June); therefore this signal may represent particulates produced from both coccoliths and organic particles during the blooms at these sites.

Dissolved oxygen ranged from 206.1 to 288.8 μmol L^−1^ across all sites and in all cases was seen to decrease with depth. Oxygen saturation ranged between 70 and 90% at the depths where the CWC reefs were located. The shallower sites did not reach as low oxygen concentrations as the deeper sites (dissolved oxygen was 250.3–286.9 μmol L^−1^ at the MRC vs. 206.1–268.2 μmol L^−1^ at Logachev), although there was still a removal of oxygen at the depths of the reefs. At the HTS, an oxygen minimum (203.6 μmol L^−1^) was observed at about 1025 m, which corresponded approximately to the depth at the top of the seamount. Below this oxygen minimum, DO increased again to 257.5 μmol L^−1^ at about 1900 m.

### Nutrients and the carbonate system

Nitrate, silicate and phosphate concentrations increased with depth at all sites (Fig. 3). Surface nitrate and phosphate concentrations were lowest at MA01 (2.2 μM and 0.3 μM, respectively) whereas at Logachev and Pisces, the concentrations were still relatively high in the surface layers (>4 μM and >0.4 μM, respectively) (Table 1).

Dissolved inorganic carbon (C_T_) increased with depth at all sites, even when normalized to salinity (nC_T_) (Fig. 4). A_T_ was generally more stable through the water column, although there was greater variation through time. When normalized to salinity (nA_T_), each site showed a slightly different pattern in alkalinity with depth (Fig. 4). At MA01 and Banana reef there was, on average, a small decrease in nA_T_ with depth (ΔnA_T_ (from surface to the depth of the reef) = ~8 μmol kg^−1^ and ~5 μmol kg^−1^, respectively). At Logachev, the water column was generally well mixed with respect to nA_T_ above 450 m, below which it began to increase from about 2285 μmol kg^−1^ to a maximum of 2320 μmol kg^−1^ at 700 m. The HTS showed a slight decrease from the surface to 250 m (~2310 μmol kg^−1^ to ~2290 μmol kg^−1^), and remained stable until below 1000 m when it began to increase again to ~2310 μmol kg^−1^ at 1500 m. At Pisces there was an increase in nA_T_ with depth, however, this appears to be driven by a decrease in alkalinity in the surface waters as the result of calcification from a coccolithophore bloom that was occurring at the time of sampling.

Formation and remineralisation of organic matter is generally assumed to occur at a C:N:P ratio (the Redfield ratio) of 106:16:1^31^. Across all sites the nC_T_:P ratio was lower than the Redfield ratio, at approximately 75:1 (Fig. 5), while the N:P ratio was much closer to the Redfield ratio at 15.7:1 (Fig. 5). Therefore again indicating an overall signal of carbon addition, although this signal is influenced by the greater sampling effort that occurred at Logachev (Fig. 5). C_T_ also increased with decreasing temperature by approximately 15 μmol kg^−1^ °C^−1^ across all sites except for Mingulay, which showed a shift in C_T_ without a significant change in temperature (Fig. 5).

Statistically, C_T_ and A_T_ profiles were significantly affected by the sites themselves (ANOSIM, *Global R* = 0.326, *p* = 0.01). However, pairwise tests revealed MA01 and Banana reef were not statistically different (*R* = −0.02, *p* = 0.57), but MA01 was most distinct from all the other sites (*p* = 0.01 in all cases). Furthermore, when depth included as a factor, MA01 is distinct from all sites (*p* < 0.012 in all cases), except Banana reef (*p* = 0.598). Between the other three sites (Logachev, Pisces and HTS), only Logachev and Pisces were statistically distinct (*R* = 0.255, *p* = 0.045).

pH_T_ ranged from 8.19 to 7.92, decreasing with depth from an average pH of >8.10 in the upper mixed layer. Ω_Aragonite_ ranged from 1.1 to 2.6 across all sites and depths; again, generally showing a decrease in saturation state with depth at all sites (Fig. 4).

nC_T_ and Ω_Aragonite_ both had strong significant correlations with oxygen % saturation. The relationships are provided here for Logachev site only; these relationships are explored in more detail in supplementary information (S2) online: 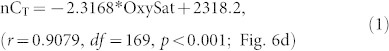

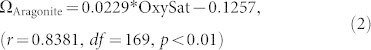


### Logachev fine-scale dynamics

**Figure 1:**
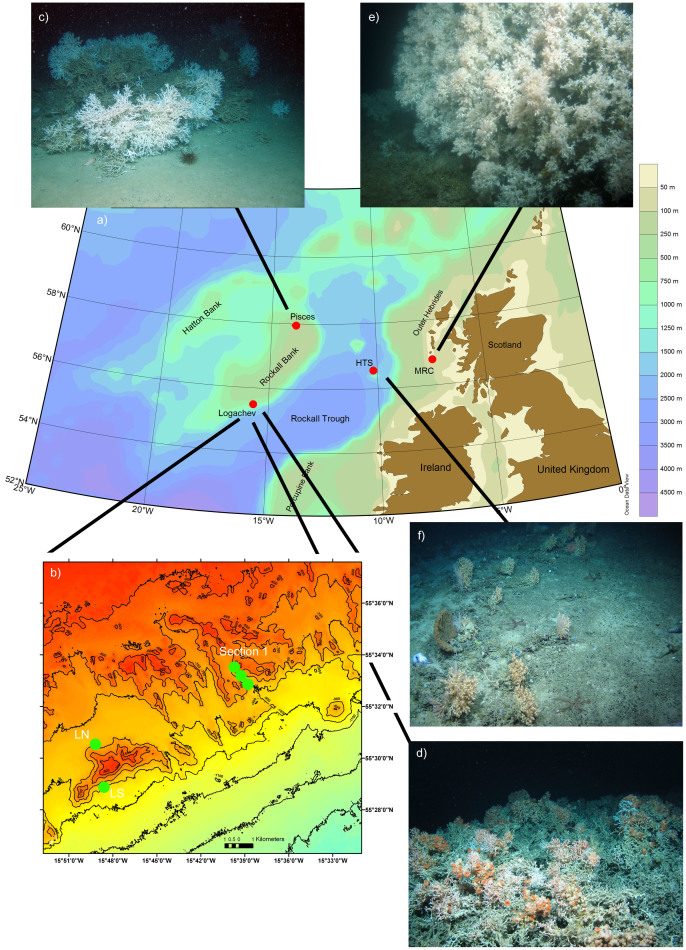
Map and example habitats of the sites studied in the northeast Atlantic during the ‘*Changing Oceans*’ expedition: (a) map (produced using ODV) showing the location of the four main sites: MRC = Mingulay Reef Complex, HTS = Hebrides Terrace Seamount, Logachev, and Pisces; (b) bathymetric map of the Logachev area (produced using ArcGIS 9, ESRI), highlighting the location of the fine-scale study areas LS = Logachev South, LN = Logachev North, and Section 1; (c) example of the ‘Wilson ring’ coral patches at Pisces; (d) example of the large coral carbonate mounds topped with large *Lophelia pertusa* structures at Logachev; (e) example of the shallower *L. pertusa* reefs at the MRC; and (f) example of the ‘coral garden' habitats some of which are structured by the colonial scleractinian *Solenosmilia variabilis* at the HTS. Photographic images taken during Changing Oceans Expedition 2012 (RRS James Cook cruise 073). Images c, d, e courtesy Heriot-Watt University. Image f courtesy Heriot-Watt University and the Joint Nature Conservation Committee.

**Figure 2:**
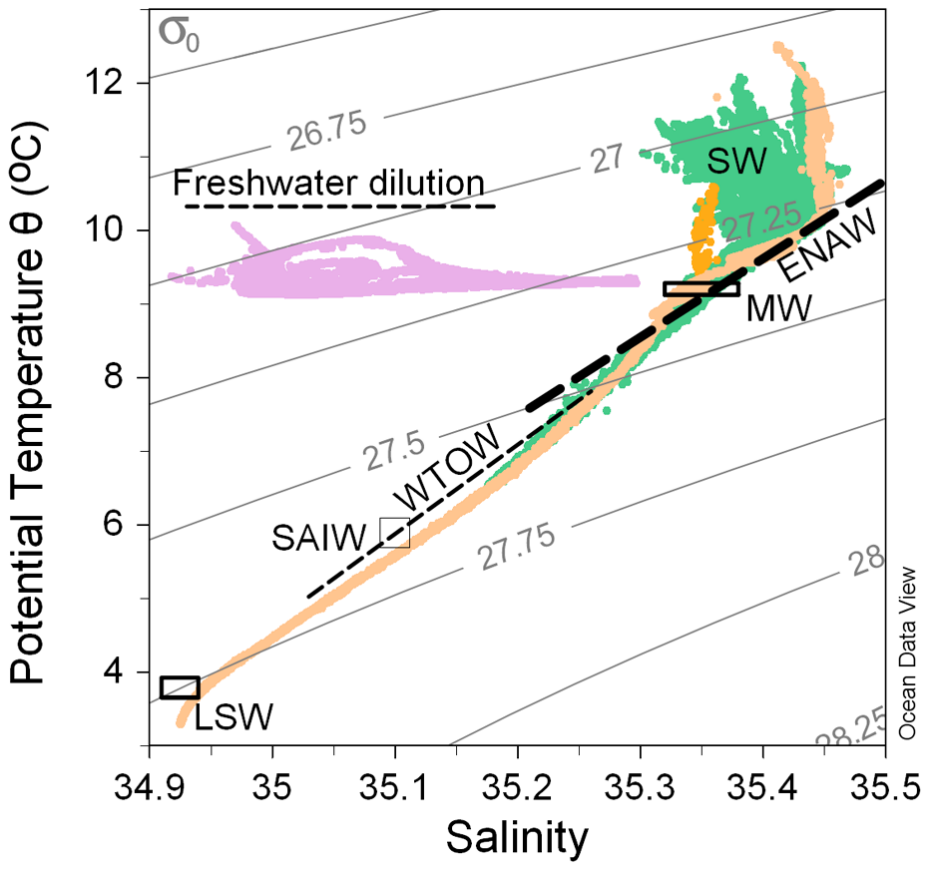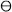-S plot with isopycnols, for the four main reef locations also showing the relevant water masses either as mixing lines (East North Atlantic Water (ENAW) and Wyville-Thomson Overflow Water (WTOW)) or boxes (Mediterranian Water (MW), Sub-Arctic Intermediate Water (SAIW) and Labrador Sea Water (LSW)), taken from McGrath et al. (2012b). Surface water (SW) and coastal freshening (freshwater dilution) are also indicated. Isopycnols are drawn in light grey. In colour, data points are marked as: Mingulay Reef Complex (MRC) = purple, Logachev mounds = green, Pisces = dark orange, and Hebrides Terrace Seamount (HTS) = pale orange.

**Figure 3:**
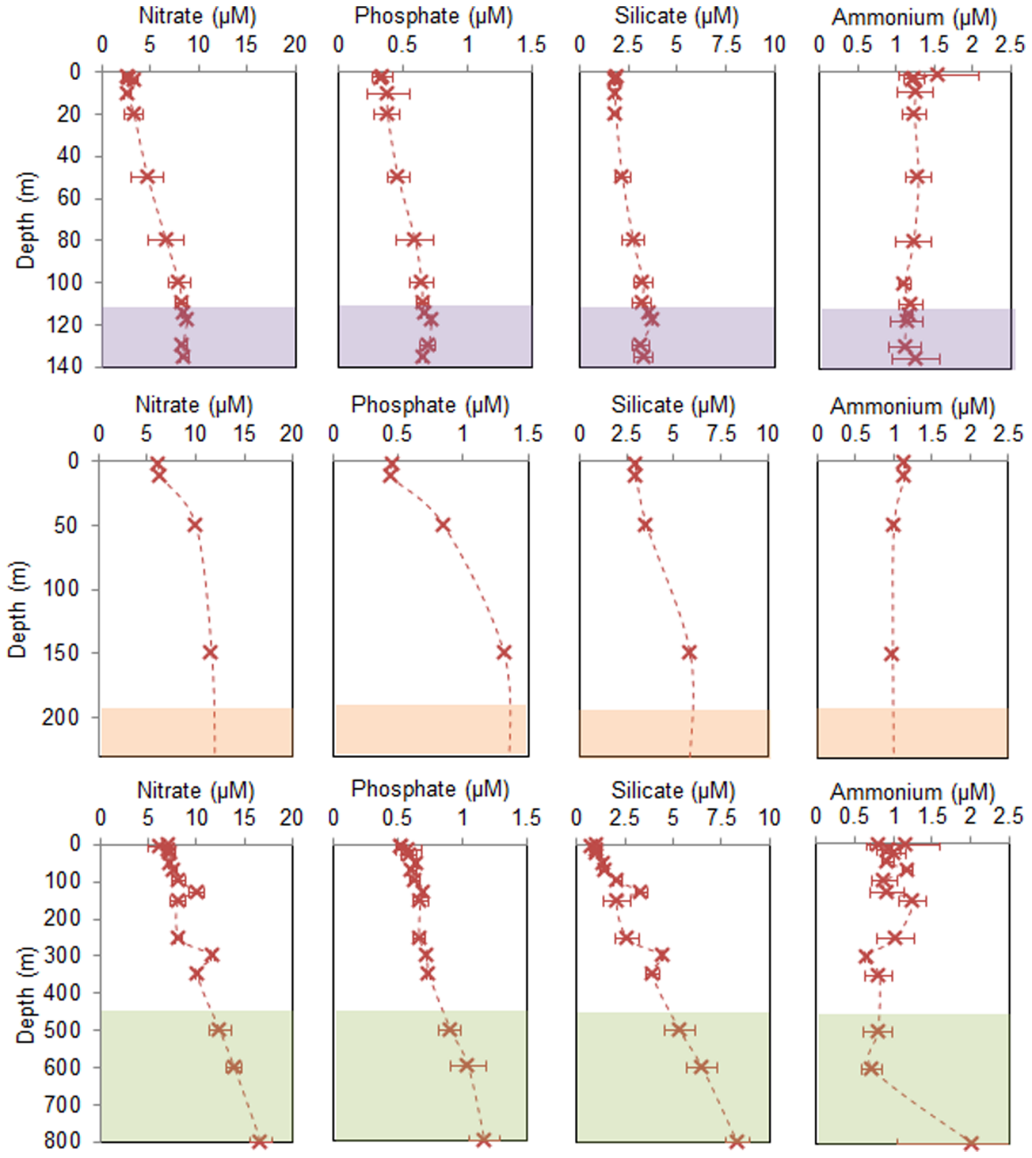
Average (mean ± standard deviation) profiles through the water column at three of the sites: (first row) Mingulay Reef Complex, (second row) Pisces, and (third row) Logachev, for (first column) nitrate (μM), (second column) phosphate (μM), (third column) silicate (μM), and (fourth column) ammonium (μM). Also showing the depth ranges where CWC reefs were observed during ROV dives (colored areas on each graph). No nutrient data were collected at the Hebrides Terrace Seamount. Note the depth scale changes.

**Figure 4:**
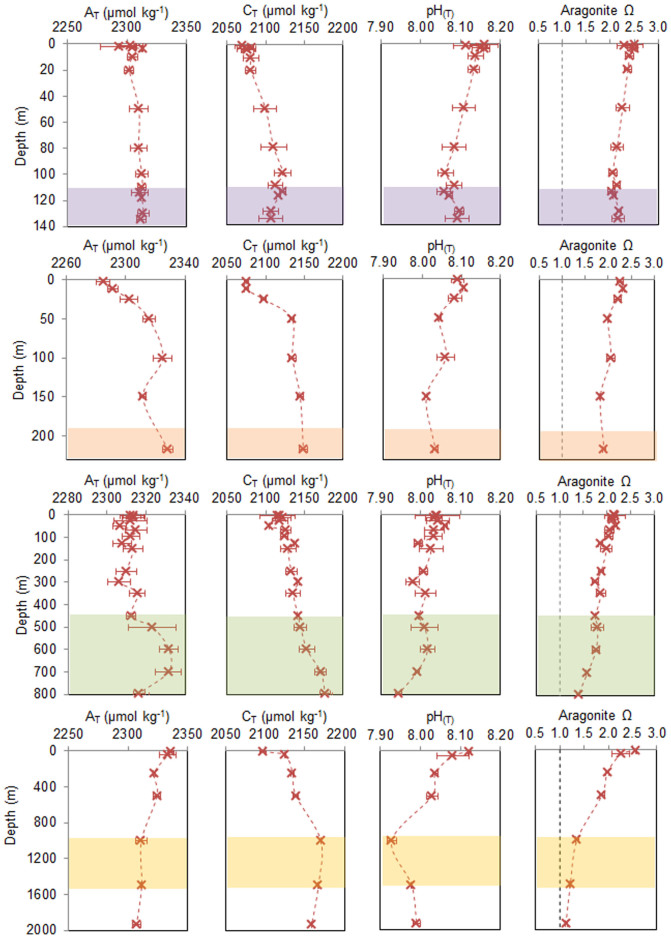
Average (mean ± standard deviation) profiles through the water column at all sites: (first row) Mingulay Reef Complex, (second row) Pisces, (third row) Logachev, and (fourth row) Hebrides Terrace Seamount, for (first column) total alkalinity (A_T_), (second column) dissolved inorganic carbon (C_T_), (third column) pH (total scale), and (fourth column) aragonite saturation state (Ω_aragonite_). Also showing the depth ranges where CWC reefs were observed during ROV dives (colored areas on each graph). Note the depth scale changes.

**Figure 5:**
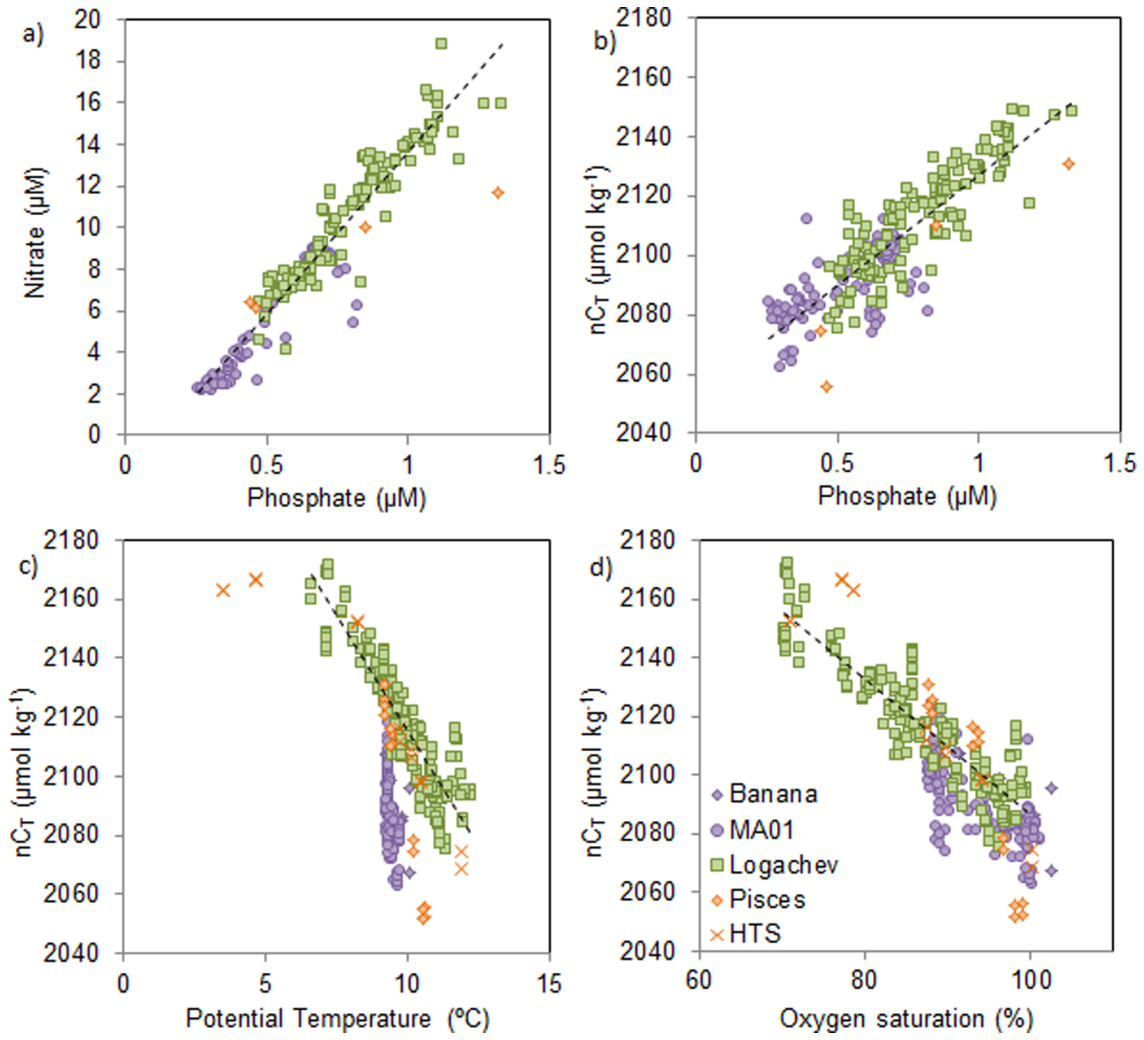
Relationships between (a) nitrate and phosphate, (b) nC_T_ and phosphate, (c) nC_T_ and potential temperature, and (d) nC_T_ and oxygen saturation (%). Coloured symbols represent the different sites (as in Figure 2); dashed black lines in each plot represents the line of best fit to the data.

**Figure 6:**
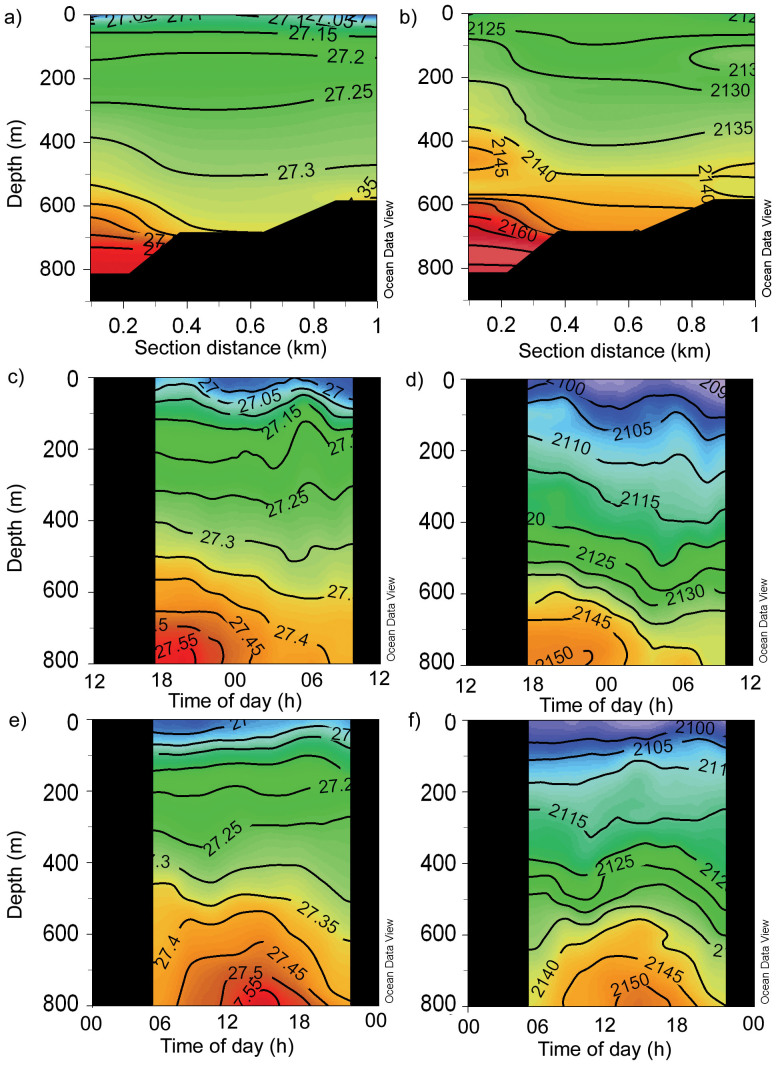
Profiles of density (a, c, e) and nC_T_ (μmol kg^−1^) estimated from Oxygen % saturation (see text, equation 1) (b, d, f) across a transect ‘Section 1’ up onto the Rockall Bank (a and c), and through time, over one 24 hour period, for stations at Logachev North ‘LN’ (c and d) and Logachev South ‘LS’ (e and f). See figure 1 for location of the transect and the two stations LS and LN.

Data from a transect (‘Section 1’ Fig. 1a) onto the Rockall Bank in the Logachev region shows the fine-scale spatial variability, and indications of potential upwelling of deeper waters by internal waves onto the carbonate mounds, where CWC are located between 800 and 600 m (Fig. 6). At both LS and LN, internal waves influence temperature and salinity most significantly below 500 m^30^, as evidenced in our dataset: At LS, the daily Δtemperature and Δsalinity at 500 m was 0.71°C and 0.06 psu; at 600 m was 0.90°C and 0.07 psu; and at 800 m was 2.05°C and 0.14 psu. At LN, the daily Δtemperature and Δsalinity at 500 m was 0.78°C and 0.06 psu; at 600 m was 0.88°C and 0.06 psu; and at 800 m was 1.77°C and 0.11 psu. Nitrate, phosphate, silicate and nC_T_ all showed corresponding changes through time in the water column below 500 m, while nA_T_ showed very little variability through a daily cycle, although fewer discrete samples for C_T_, A_T _and nutrients were taken compared to the CTD data. For example, at both LS and LN, at the depth of 600 m, nC_T_ varied by ~40 μmol kg^−1^ through the 12 h period, while nA_T_ varied by only 14 μmol kg^−1^. The change in C_T_ caused a change in the other carbonate system parameters, for example aragonite saturation state varied by ~0.2 over the period. Because of the strong relationships with oxygen saturation (%), and the more frequent data available from the oxygen sensor mounted on the CTD, this relationship (Eq. 1) was used to calculate full profiles for nC_T_ over the 12 h periods at the two Logachev sites (Fig. 6).

## Discussion

Empirical observations for nutrient and carbonate system dynamics are shown for a number of CWC habitats: one coral carbonate mound, two cold-water coral (CWC) reefs, and one Seamount found in the northeast Atlantic region (Fig. 1). The dissolved inorganic carbon (C_T_) and alkalinity (A_T_) concentrations are within 1.5% and 1% respectively of data extracted from the GLODAP database (comparing GLODAP gridded data at 55.5 N, 15.5 W^16^ with mean values from Logachev at 55.5 N, 15.7 W).

The environmental conditions at the study sites predominantly fall within the global envelope for CWCs described in Davies *et al.*^5^. The nutrient concentrations fall within the range prescribed for the NE Atlantic^5^; however, here we provide data for the carbonate system parameters that were not previously available at a high resolution of sampling. Flögel *et al.*^19^ recently published a comparison of available carbonate system data for CWC in the North Atlantic, including the Mediterranean, which fits well with our data collected at similar locations, and supports earlier data from this region [e.g.^11^,^32^]. Flögel *et al.*^19^ suggest coral “quality” is associated with a seawater density range similar to that originally described by Dullo et al.^4^ and highlighted in niche width models by Davies *et al.*^5^, but also that low quality CWCs are exposed to C_T_ concentrations <2170 μmol kg^−1^.

A number of recent publications provide a wider comparison of carbonate and nutrient conditions across sites of known CWC reefs, between the NE Atlantic [our data^5^,^19^], the Mediterranean, the Gulf of Cadiz, and Mauritania^11^,^19^,^32^, the Gulf of Mexico (GoM)^18^, Chilean fjords^32^,^33^, the Marmara Sea and the Tasman Seamount^32^ (Table 2). CWC reefs in the NE Atlantic (excluding the Mediterranean) appear to experience slightly lower C_T_ concentrations than CWC reefs in the GoM, but similar levels of A_T_, which results in the GoM experiencing, on average, lower Ω_Aragonite_ than in the NE Atlantic (Table 2). Although no assessment of CWC quality is provided by Lunden *et al.*^18^, C_T_ is higher than the proposed limit of 2170 μmol kg^−1^^19^. Furthermore, Lunden *et al.*^18^ found no significant relationship between skeletal density and Ω_Aragonite_, which suggests these conditions were not limiting for calcification. The Chilean fjords have similar and slightly lower levels of C_T_ but significantly lower salinity and consequently significantly lower A_T_, and again therefore experience lower Ω_Aragonite_ than the NE Atlantic (Table 2). However, different coral species (*Desmophyllum dianthus*) are abundant at the Chilean sites and this also needs to be taken into account when assessing CWC sensitivities.

While these datasets provide just one or two data points for each location (for an average or “spot” condition), we highlight here that measuring through time at one site provides as much variability as is found across the sites (Table 1 and Table 2). For example, at MA01, the localised downwelling caused C_T_ to decrease below the lowest reported value from Flögel *et al.*^19^, to <2090 μmol kg^−1^, with a total C_T_ range at MA01 of 40 μmol kg^−1^. At the Logachev site, upwelling and internal waves caused C_T_ to increase above the highest reported value from Flögel *et al.*^19^, to >2185 μmol kg^−1^. Across the Logachev site we found a total C_T_ range of 58 μmol kg^−1^, which is nearly double the reported range for the Western Rockall Bank (24 μmol kg^−1^^19^,) for CWC given a quality category of CI-CII^19^. Interestingly, the majority of CWC reefs attributed to quality category CIII^19^ were found predominantly in the Mediterranean, which naturally has high temperatures and salinities, but additionally in Mauretania and in the Gulf of Cadiz, both of which also have higher temperatures than the majority of CI and CII sites^19^.

pH_T_ and Ω_Aragonite_ varied by approximately 0.27 and 1.51 respectively (Table 1), over the spatial (and depth) region investigated here, although the hydrodynamics clearly increased the variability for pH_T_ and Ω_Aragonite_ at the sites where finer time-scale measurements were carried out (MA01, LS and LN; Table 1). Indeed, using *in situ* lander measurements, Mienis *et al.*^30^ described the fine-scale dynamics at the southwest Rockall Trough margin (Logachev region) over a longer sampling period (about 1 year) and found that the water current speeds peaked just before or at the same time as the peaks in temperature and salinity. The physical conditions (temperature and salinity) in our dataset correspond to the observations of Mienis *et al.*^30^, where internal waves cause a diurnal shift in the physical conditions. The transport of C_T_ and nutrients, along with sediments and particles *via* these internal waves could prevent a build-up of high CO_2_, low oxygen conditions around the CWC reefs, flushing them with fresh material, nutrients and lower CO_2_ conditions over a diurnal cycle. Therefore on a fine-scale these hydrodynamics create additional variability not accounted for by cruises that only sample once per station. Indeed Flögel *et al.*^19^ suggest a narrower range of conditions could be considered if Mingulay Reef is excluded from their assessment because of its downwelling impacts, yet the very nature of this downwelling contributes to a higher variability in chemical components such as C_T_ and nutrients, and consequently impacts the local environment for CWCs and associated biodiversity^29^.

Kenyon *et al.*^24^, suggested that CWC reefs in the North Atlantic were found in the Oxygen Minimum Zone (OMZ). An OMZ usually corresponds with high C_T_ and pCO_2_ because OMZs arise from remineralisation of organic matter and therefore consumption of oxygen, and release of CO_2_. Our dataset shows a strong negative relationship between oxygen % saturation and nC_T_, such that at the lowest oxygen levels there was highest nC_T_ concentration (e.g. nC_T_ ≈ 2160 μmol kg^−1^ at 70% oxygen saturation). Correspondingly, these low DO areas also exhibit the lowest pH and low Ω_Aragonite_ (e.g. at 70% oxygen saturation, pH ≈ 7.94 and Ω_Aragonite_ ≈ 1.3, although Ω_Aragonite_ continued to decrease with depth after the OMZ because of the influence of low saturation state in the LSW). CWC reefs therefore appear to be thriving in conditions that are conventionally considered unsuitable for many organisms, particularly calcifiers, and this confirms that the variable physicochemical environment found here is not limiting CWC reef growth. The natural fluctuations in the physicochemical conditions (including oxygen, carbon, nutrients and food supply), driven by large hydrodynamics, may provide flexibility for these organisms, giving them increased adaptation potential for surviving a range of conditions, as has been found with warm-water corals and temperature^34^. This naturally fluctuating environment could help to explain the high levels of variability found in CWCs ocean acidification experiments, especially as several recent laboratory experiments CWCs are able to continue calcifying under high CO_2_, low Ω_Aragonite _conditions [e.g.^35^,^36^,^37^].

Interestingly, the carbonate mounds (between 400 and 700 m water depth) at Logachev appear to provide an additional, albeit relatively small, carbon (alkalinity) source to the water column, compared to the other sites that consist of coral habitats but do not have carbonate mounds present. On average, nC_T_ steadily increased over this depth range at Logachev yet normalized alkalinity remained the same, hence the seawater pH was somewhat buffered and therefore also remained relatively uniform across the depth range of the mound. The caveat to this interpretation is the different end-members of C_T_ and A_T_ associated with different water masses; given the complex physical oceanography and diversity of water masses and potential mixing in this area, further work is required to elucidate if this apparent alkalinity increase is really from the carbonate mounds or if it is from high alkalinity water masses, for example flowing south from the Arctic, which has a different set of implications in the context for global climate change and ocean acidification.

Carbonate system measurements from the deepest samples taken at Logachev and at the Hebrides Terrace Seamount, show that the aragonite saturation state approaches 1.2–1.3 across the Rockall Trough at approximately 1000 m, decreasing to near 1.0 at 1500 m. The present day depth of the aragonite saturation horizon (ASH) in the NE Atlantic is therefore similar, or in localised areas shallower, than in model projections for the North Atlantic (e.g. the 1994 depth was approximately 2500 m^38^). Future model projections for the ASH show it shoaling to 600–800 m by mid-century (2046–2065) and then further surface-ward to around 200 m by the end of century (2080–2099)^38^. Indeed, McGrath *et al.*^17^, show that C_T_ has increased, resulting in an equivalent decrease in pH of 0.040 ± 0.003 units in the sub-surface waters, over the 19 years that were investigated for the Rockall Trough region. This was concomitant with a decrease in saturation state of both aragonite and calcite and a shoaling of the ASH.

While it appears CWC reefs are naturally exposed, and thereby appear tolerant to, a wide range of conditions, the deeper reefs could be at risk of dissolution or be affected by a shift in their physiology as a result of hypercapnia^39^,^40^. Even if the corals themselves have a degree of acclimation in response to high CO_2_ conditions^32^,^35^,^36^,^37^,^39^,^40^, dissolution of the coral carbonate mounds, such as those found at Logachev, and any dead coral structure, which provides the hard substrate on which corals and abundant epifauna can grow^41^, could become unstable as the ASH shoals. Coupled with predicted increased efficiency of bio-eroding sponges^42^ and a multitude of additional threats^43^, these deeper reefs could face significant threat of degradation. Furthermore, the differing rates of acidification occurring in the different water masses that are associated with these reefs (e.g. increased acidification rate in LSW compared with surface waters^17^) will also alter the regional threat to these important communities.

## Methods

A total of 30 CTD and rosette sampling casts were carried out across the sites (MRC, Logachev, Pisces and HTS (Fig. 1)) between 21^st^ May and 9^th^ June 2012^44^,^45^ during the ‘Changing Oceans Expedition 2012’, on board the RRS *James Cook*, cruise JC073^46^. A Sea-Bird 911 plus CTD system (9*plus* underwater unit and Sea-Bird 11*plus* deck unit) was deployed with a Rosette water sampling unit, fitted with 10 L Niskin water bottles. Water samples were taken at discrete depths throughout the water column with the deepest samples being taken approximately 2 m above the reef (using altimeter information on-board the CTD). These samples are referred to as “immediately above the reef”. Pre-cruise laboratory calibrations were performed on the conductivity, temperature and pressure sensors, all giving coefficients for linear fit. Also attached to the Rosette was a Sea-Bird 43 dissolved oxygen sensor, a Chelsea Aquatracka MKIII fluorometer (set to detect Chlorophyll α: excitation wavelength of 430 nm and emission wavelength of 685 nm), and a Chelsea Aquatracka MKIII Transmissometer, which were used to measure dissolved oxygen, chlorophyll fluorescence, and particle attenuation coefficient (*Cp*; measured at wavelength of 660 nm; e.g. Behrenfeld and Boss^47^, and references therein), respectively. Dissolved oxygen was calibrated against Winkler titrations^48^ made on discrete water samples collected from a range of depths, and produced an offset between the titrations and sensor measurements of <1%. Because of the fine-scale of the hydrodynamics being assessed here, the up-cast raw data were used for the analysis and interpretation of the discrete measurements to match water column state at the time of bottle firing (see supplementary information S3 online for up-cast vs down-cast variability assessment). Further data processing was performed using the software SBE Data Processing (V7.21g) and for data visualization Ocean Data View (V4.3.7)^49^ was used.

Seawater was collected from the Niskin bottles for the dissolved inorganic carbon (C_T_) and total alkalinity (A_T_) analysis. Samples were collected in borosilicate glass bottles with ground glass stoppers (50 mL), which were rinsed and filled according to standard procedures detailed in^50^. Samples were poisoned with 10 μL mercuric chloride (HgCl_2_) and duplicate samples were taken from the same Niskin bottle. Samples were returned to the chemical laboratory onboard the RRS *James Cook*, where they were normalized to room temperature (approx. 24°C) and analyzed for C_T_ and A_T_ within 24 hours of collection.

C_T_ was measured using a Dissolved Inorganic Carbon Analyzer (Apollo SciTech, Model AS-C3) calibrated using CO_2_ Certified Reference Materials (Dickson, Batch 113). Duplicate measurements provided an estimate of measurement error, which was 0.2% across the entire dataset. An assessment of errors for each site is provided in supplementary information S4 online. C_T_ was corrected for the addition of HgCl_2_.

A_T_ was measured using the open-cell potentiometric titration method using an automated titrator (Apollo SciTech Alkalinity Titrator Model AS-ALK2). Calibration was made using CO_2_ Certified Reference Materials (Dickson, Batch 113). Duplicate measurements were made for each sample, and the estimate of measurement error was 0.4% across the entire dataset. An assessment of errors for each site is provided in supplementary information S4 online. A_T_ was corrected for the addition of HgCl_2_.

Seawater was collected for nutrient analysis from the CTD Niskin bottles directly after samples were taken for the carbon analysis. 50 mL of collected seawater was filtered (0.45 μm acid-washed Millipore Fluoropore) into acid-cleaned, aged, 60 mL Nalgene bottles, duplicate samples were collected from each Niskin. Bottles were stored and shipped back frozen (−20°C) to Plymouth Marine Laboratory, where analysis was carried out^51^ using a Bran and Luebbe AAIII segmented flow autoanalyzer for the colorimetric determination of inorganic nutrients: combined nitrate and nitrite^52^, nitrite^48^, phosphate^53^, and silicate^54^. Nitrate concentrations were calculated by subtracting nitrite concentration from the combined nitrate + nitrite concentration.

The remaining carbonate system parameters (pH_T_, pCO_2_, Ω_aragonite_) were calculated from measured A_T_ and C_T_, together with depth, temperature, salinity, silicate, and phosphate (when available), using the programme CO2sys^55^, with dissociation constants from^56^ refit by^57^ and for KSO_4_ from^58^.

In addition to temperature and salinity, three other major processes influence carbonate chemistry: organic matter formation and remineralisation, calcium carbonate calcification and dissolution, and air-sea gas exchange. At the depth of the CWC reefs described here, air-sea gas exchange is assumed to be negligible; therefore we assume that air-sea gas exchange only contributes to carbon dynamics in the surface waters. To remove the effects of salinity, C_T_ and A_T_ were normalized to a reference salinity (*S^ref^* = 35) using standard methods (see^59^ for discussion of normalization options) and are denoted as nC_T_ and nA_T_, respectively. For example, for measured A_T_ (*A_T_^meas^*) and measured salinity (*S^meas^*): 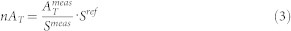


At the two Logachev stations LS and LN, CTD profiling was conducted continuously for a period of just over 12 hours at each site (first at LS and then at LN); with additional discrete samples taken at 500 m and 600 m: four times at (LS) and six times at (LN). At this site the deepest samples were taken immediately above the coral carbonate mounds (see supplementary Fig. S7 online). An ROV survey of the top of the carbonate mound revealed *Lophelia* reefs on the top of the mound.

An Analysis of Similarities (ANOSIM) test was conducted to assess similarities in the carbonate system and nutrients across the different sites, using Primer v6^60^. Correlations between variables was analyzed for statistical significance using Pearsons correlation coefficients, and in some cases the slopes were used to test for significant differences between regression lines.

## Author Contributions

H.S.F., S.J.H., L.C.W. and J.M.N. collected the data. H.S.F. and E.M.S.W. analyzed the data. H.S.F. wrote the paper, and all authors (H.S.F., S.J.H., J.M.N., L.C.W., E.M.S.W. and J.M.R.) contributed to the final text and figures.

## Supplementary Material

Supplementary Information Supplementary Inforamtion

## Figures and Tables

**Table 1:** Range (minimum – maximum) of environmental conditions observed at the each site through the whole water column: Mingulay Area 01 (MA01), Banana Reef, Logachev, Pisces, and Hebrides Terrace Seamount (HTS). pH_T_ and Ω_arag_ were calculated from C_T_ and A_T_, salinity, temperature, phosphate and silicate (when available), using CO2sys. C_T_ and A_T_ have been normalised (nC_T_ and nA_T_) to salinity of 35, to compare across sites. nd = no data available

	Site				
	MA01	Banana	Logachev	Pisces	HTS
Dates	21–23/05/12	24/05/12	26/05–06/06/12	07/06/12	09/06/12
Max. depth (m)	135	130	910	220	1930
nC_T _(μmol kg^−1^)	2062–2124	2067–2110	2075–2172	2052–2131	2068–2168
nA_T _(μmol kg^−1^)	2266–2321	2298–2317	2263–2321	2258–2308	2289–2319
pH_T_	8.03–8.19	8.06–8.14	7.94–8.10	8.01–8.11	7.92–8.13
Ω_aragonite_	1.92–2.62	2.05–2.42	1.35–2.35	1.82–2.34	1.11–2.58
HCO_3_^−^ (μmol kg^−1^)	1878–1994	1908–1969	1925–2058	1905–2004	1910–2043
CO_3_^2−^ (μmol kg^−1^)	130–173	135–160	103–157	123–155	105–170
NO_3 _(μmol L^−1^)	2.18–9.00	nd	4.10–18.82	6.14–11.65	nd
NO_2_ (μmol L^−1^)	0.04–0.43	nd	0.02–0.31	0.04–0.44	nd
PO_4_ (μmol L^−1^)	0.26–3.59	nd	0.48–3.04	0.44–1.31	nd
SiO (μmol L^−1^)	1.67–3.74	nd	0.68–9.40	3.01–5.87	nd
DO (μmol L^−1^)	250.3–286.9	255.3–288.8	206.1–268.2	251.2–274.9	207.2–270.8
Fluorescence (μg Chl L^−1^)	0.04–0.50	0.05–0.30	0.02–0.37	0.04–0.87	0.02–1.17

**Table 2:** The range (published min-max) of environmental conditions at different regions, specifically at the depth and site of known CWC reefs, comparing data from this study with data available in the literature^5^,^11^,^19^,^32^,^33^

Location	NE Atlantic (this study)	NE Atlantic	Gulf of Cadiz	Mauretania	Mediterranean	Gulf of Mexico	Chilean Fjords	Marmara Sea	Tasman Seamount
Depth (m)	120–1000	100–950	606–1322	451–568	89–850	307–620	20–200	932	1050
Temperature (°C)	7.2–10.0	5.9–10.6	9.2–11.0	9.7–11.7	12.8–16.8	nd	10.6–12.5	14.5	4.59
Salinity	35.1–35.4	35.0–35.7	35.6–36.0	35.2–35.4	37.8–38.8	35.05*	31.7–33.0	38.8	34.4
A_T_ (μmol kg^−1^)	2299–2346	2287–2377	2332–2342	2314–2375	2520–2742	2259–2391	2136–2235	2610	2315
C_T_ (μmol kg^−1^)	2088–2186	2118–2174	2180–2200	2183–2240	2226–2349	2135–2231	2025–2188	2470	2218
Ω_Aragonite_	1.35–2.44	1.39–3.03	1.43–1.83	1.31–1.58	2.59–4.06	1.19–1.69	0.78–1.60	1.46	1.02
C-refs.		19	19	19	11,19,32	11	32,33	32	32
PO_4_ (μmol L^−1^)	0.6–1.5	0.4–1.6			0.20–0.41				
NO_3_ (μmol L^−1^)	4.1–18.8	8.0–23.4			nd				
NH_4_ (μmol L^−1^)	0.5–1.6	nd			0–0.29				
SiO (μmol L^−1^)	2.1–9.4	2.2–46.6			nd				
N.-Refs.		5			11				

C-Refs. = References for carbon system data; N-Refs. = References for nutrient data; nd = no data reported.

*Only mean salinity reported in^11^.
