# Supplementary material for: Fine-scale nutrient and carbonate system dynamics around cold-water coral reefs in the northeast Atlantic
Source: Sci Rep. 2014 Jan 20;4:3671. doi: 10.1038/srep03671 (PMC3895924; doi:10.1038/srep03671)
Supplement: Supplementary Information — Supplementary Inforamtion [file srep03671-s1.doc]

**Fine-scale nutrient and carbonate system dynamics around cold-water coral reefs in the northeast Atlantic - Supplementary Information**

Helen S. Findlay, Sebastian J. Hennige, Laura C. Wicks, Juan Moreno Navas, E. Malcolm S. Woodward, J. Murray Roberts

**Contents:**

There are 4 sections in this Supplementary information:

S1: Fluorescence (chlorophyll) & particle attenuation coefficient (Cp)

S2: nC_T_ and Ω_Aragonite_ correlations with oxygen saturation (%)

S3. CTD down-cast vs. up-cast

S4: Carbonate system measurement errors

S5: Location of the LS and LN sites

S1. Fluorescence (chlorophyll) & particle attenuation coefficient (*Cp*)

**Correlations between Chlorophyll Fluorescence and particle attention coefficient (*Cp*) were investigated across all sites (Table S1), and at each site independently (Fig. S1). Comparison between regression lines was conducted to assess whether the relationship between Florescence and *Cp* differed between sites (Table S2 and S3).**

**All sites together:**

***Cp* = 0.673*Fluorescence + 0.1277**

S = 0.0970028 R^2^ = 25.9% R^2^(adj) = 25.9% r = 0.51

Table S1: Analysis of Variance.

| Source | DF | SS | MS | F | P |
| --- | --- | --- | --- | --- | --- |
| Regression | 1 | 357.57 | 357.57 | 38000.41 | 0.000 |
| Residual Error | 108577 | 1021.66 | 0.01 |  |  |
| Total | 108578 | 1379.23 |  |  |  |

**Table S2: Slope, standard error (SE) and number of measurements (n) for the regression line between Fluorescence and *Cp* at each of the sites.**

|  | MA01 | HTS | Logachev | Pisces |
| --- | --- | --- | --- | --- |
| b | 0.6437 | 0.8143 | 0.3724 | 1.946 |
| SE | 0.039477 | 0.069193 | 0.010552 | 0.08637 |
| n | 2750 | 7470 | 94136 | 875 |

**Table S3: Results of t-test for differences between regression lines, showing *t* values and probability (*p*) values.**

| *t*  *p* | MA01 | HTS | Logachev | Pisces |
| --- | --- | --- | --- | --- |
| MA01 |  | -2.1415 | 6.6392 | -13.7136 |
| HTS | 0.0323 |  | 6.3135 | -10.2261 |
| Logachev | <0.001 | <0.001 |  | -18.0848 |
| Pisces | <0.001 | <0.001 | <0.001 |  |


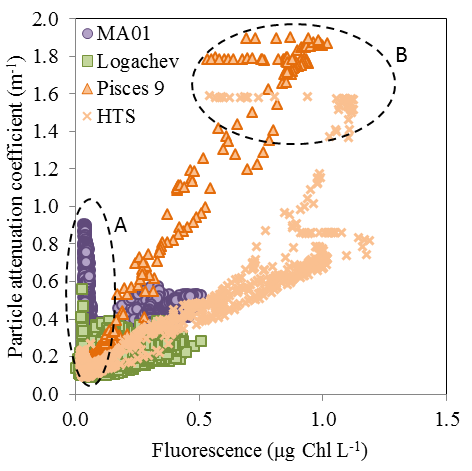


Figure S1: Fluorescence – particle attenuation coefficient scatterplot, with coloured symbols representing the different sites (as in Figure 2). Ovals marked A illustrate the particles coefficient near the reef, while oval marked B illustrate the surface particles at Pisces and HTS which represent coccolithophore bloom.


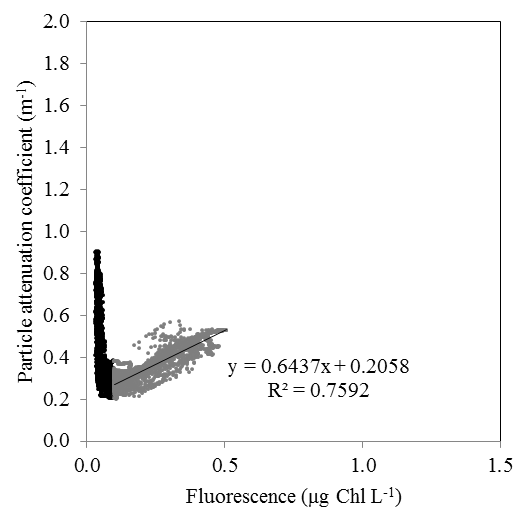

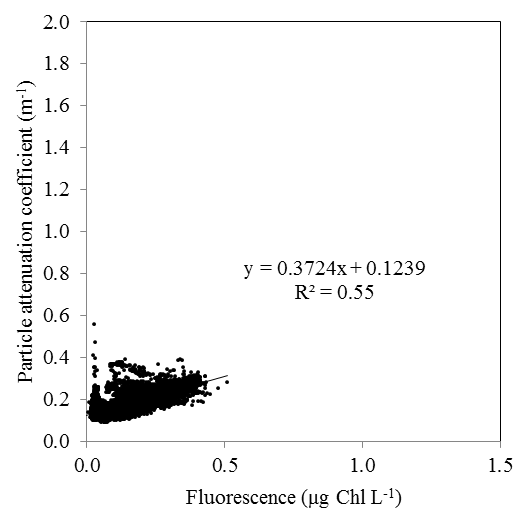

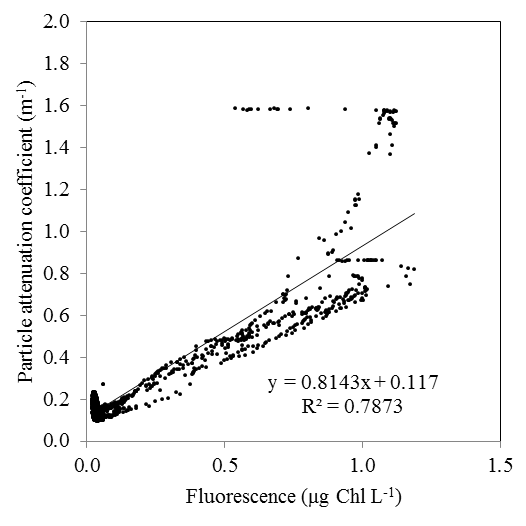

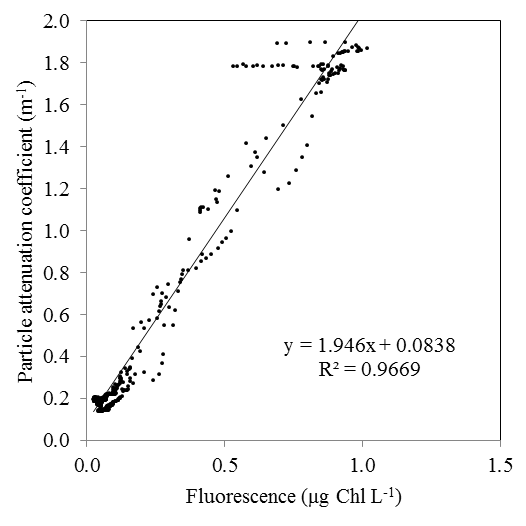


Figure S2: Fluorescence – particle attenuation coefficient scatterplots for each of the sites individually (a) MA01, (b) Logachev, (c) Hebrides Terrace Seamount, and (d) Pisces. Regression lines of best fit are shown in each plot (grey line).

S2. nC_T_ and Ω_Aragonite_ correlations with oxygen

Regression analysis was conducted on the relationship between salinity-normalised dissolved inorganic carbon (nCT) and oxygen saturation (%), and aragonite saturation state (Ω_aragonite_) and oxygen saturation (%) for all sites together and for individual sites, however we present only the Logachev results here, as we utilise these relationships in the fine-scale sampling locations (LS and LN). Results of the regression analysis are presented below together with figures of the relationships and of the measured vs. calculated parameters.

**Regression Analysis**

The regression equation is: nC_T_ = 2317 - 2.31 Oxy%sat

S = 9.15590 R^2^ = 82.3% R^2^(adj) = 82.2% r = 0.91

Table S4: Analysis of Variance.

| Source | DF | SS | MS | F | P |
| --- | --- | --- | --- | --- | --- |
| Regression | 1 | 65434 | 65434 | 780.55 | 0.000 |
| Residual Error | 168 | 14084 | 84 |  |  |
| Total | 169 | 79518 |  |  |  |


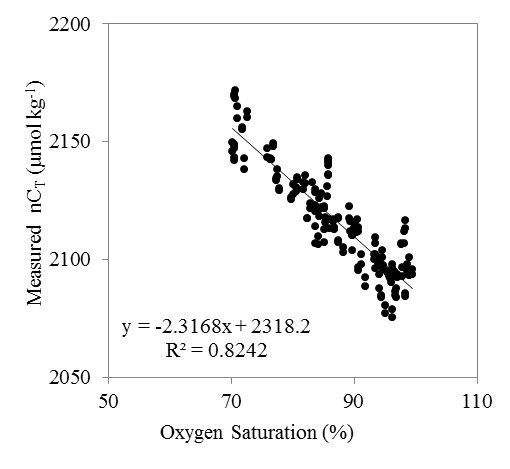

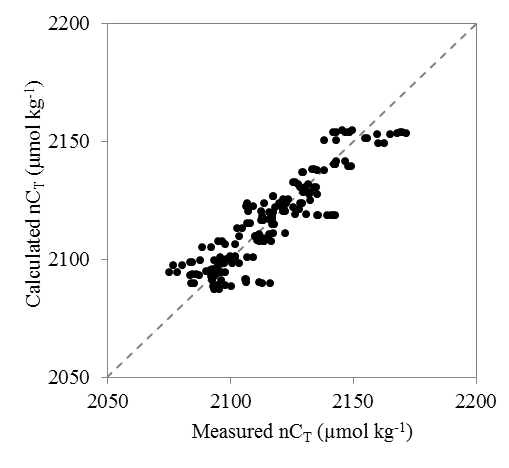


Figure S3: (a) relationship between salinity-normalised dissolved inorganic carbon (nC_T_) and Oxygen saturation (%) and (b) relationship between measured nC_T_ and calculated nC_T_ (using oxygen saturation %).

The standard error for the regression between Oxy%sat and nC_T_ is 9.156. The standard error between the measured and calculated nC_T_ is 8.2881.

**Regression Analysis**

The regression equation is: Ω_aragonite_ = - 0.126 + 0.0229 Oxy%sat

S = 0.127558 R^2^ = 70.2% R^2^(adj) = 70.1% r = 0.84

Table S5: Analysis of Variance

| Source | DF | SS | MS | F | P |
| --- | --- | --- | --- | --- | --- |
| Regression | 1 | 6.4901 | 6.4901 | 398.87 | 0.000 |
| Residual Error | 169 | 2.7498 | 0.0163 |  |  |
| Total | 170 | 9.2398 |  |  |  |


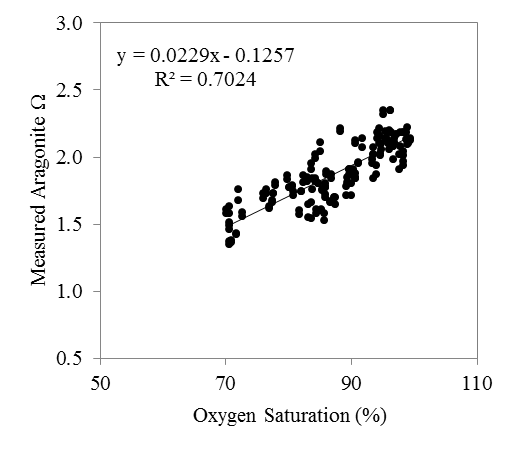

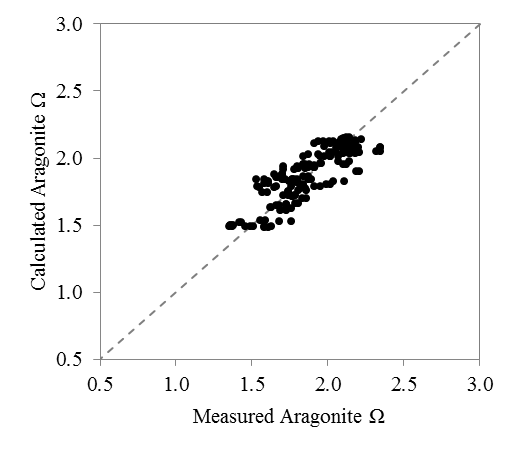


Figure S4: (a) relationship between aragonite saturation state (Ω) and Oxygen saturation (%) and (b) relationship between measured Ω and calculated Ω (using oxygen saturation %).

The standard error for the regression between Oxy%sat and Ω is 0.128. The standard error between the measured and calculated Ω is 0.1276.

**S3. CTD down-cast vs. up-cast**

In many instances down-cast data is utilised in analysis from cruise because of the potential for the CTD and instrumentation to cause turbulence and therefore disrupt the water column structure as it descends. In this paper we use the up-cast CTD data at the depths matching the bottle firing on the way up, to gain a more accurate representation of the water column at the time of the water sample. If the CTD does cause turbulence, the water collected in the bottles will be representative of that turbulent water, and not the water measured on the down-cast. Additionally, in these relatively dynamic environments we would expect that the variability caused by the CTD turbulence would be relatively small. To assess this, we have investigated the RSD of temperature and salinity data from several down- and up-casts from the CTD at corresponding (binned) depths, but made at the same location. Two examples are used here: at Mingulay and at Logachev.

At the same location at Mingulay, temperature and salinity measurements had <0.2 % RSD between up and down casts between 60 m and the bottom (~ 120 m) and made within <8 minutes of each other (Fig. S5). Between the surface and ~60 m, the salinity data was still < 0.2 % RSD between up and down casts measurements, however temperature RSD increased to a maximum of about 2.5 % between up and down cast measurements between 30 and 50 m, which represents the depth of the pycnocline, and this depth is known to vary with the tidal cycle.


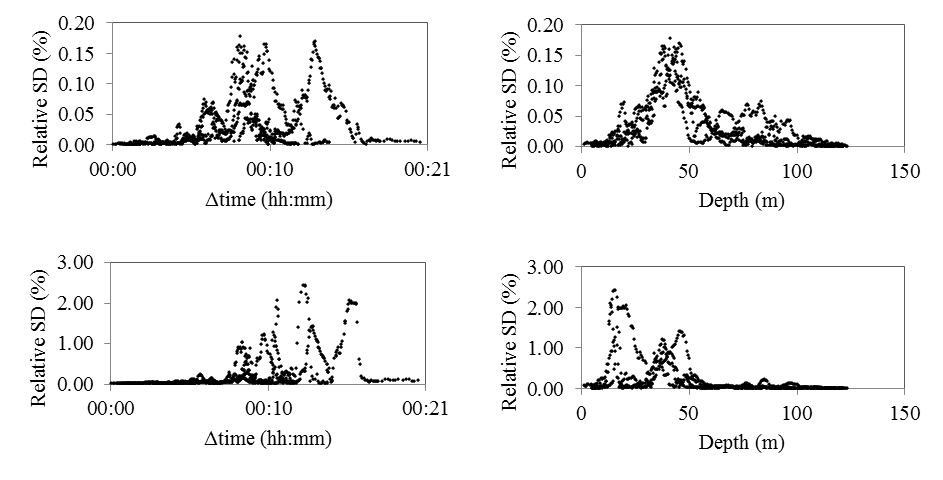


Figure S5: Data from Mingulay: Relative standard deviation (%) for salinity (top panels) and temperature (bottom panels) against time (elapsed time between measurements) and against depth (m).

At Logachev, salinity RSD was always ≤0.1%, but the difference between up- and down-cast data was least near the bottom, and least when measurements were made closer in time (Fig. S6). Temperature RSD was more variable, but again was least at the deeper sites, and least when measurements were made closer in time (Fig. S6). Even if all this variability was due to the CTD turbulence, the standard deviation, especially with salinity, is less than 0.1 %. However, we would expect that, especially at Logachev, longer periods of time between measurements would allow the water column to be restored and thus variability would be lower. This is not the case.


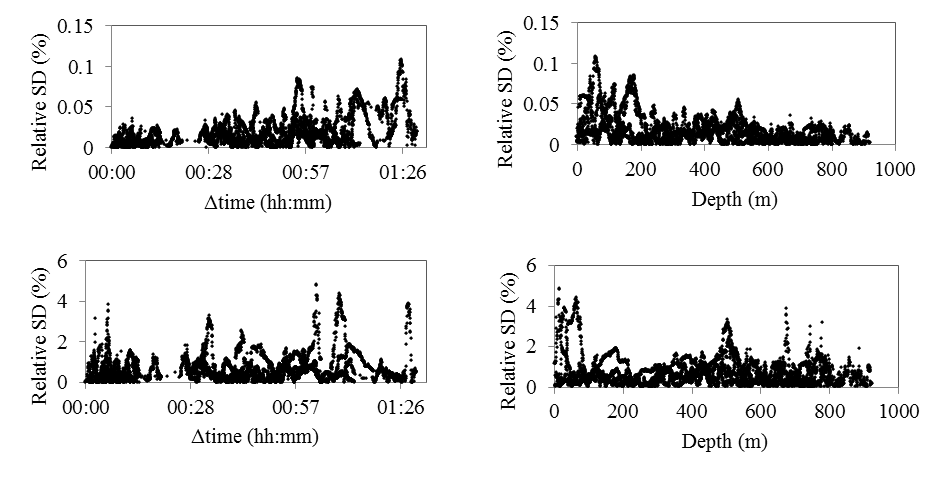


Figure S6: Data from Logachev: Relative standard deviation (%) for salinity (top panels) and temperature (bottom panels) against time (elapsed time between measurements) and against depth (m).

S4. Carbonate system measurement errors

**The average Relative Standard Deviation (RSD) for the dissolved inorganic carbon (C_T_) and total alkalinity (A_T_) measurements made across all depths and times have been calculated for each site:**

**RSD = standard deviation/mean *100**

***MA01***

**A_T_ = 0.21 %, ~4.9 µmol kg^-1^;**

**C_T_ = 0.24 %, ~5.0 µmol kg^-1^**

***Banana reef***

**A_T_ = 0.16 %, 3.7 µmol kg^-1^;**

**C_T_ = 0.34 %, ~7.2 µmol kg^-1^**

***HTS***

**A_T_ = 0.19 %, ~4.4 µmol kg^-1^;**

**C_T_ = 0.1 0%, ~2.2 µmol kg^-1^**

***Logachev***

**A_T_ = 0.17 %, ~3.9 µmol kg^-1^;**

**C_T_ = 0.14 %, ~3.1 µmol kg^-1^**

***Pisces***

**A_T_ = 0.26 %, ~6.1 µmol kg^-1^;**

**C_T_ = 0.23 % ~4.8 µmol kg^-1^**

**Therefore the observed decrease of 8 µmol kg^-1^ in A_T_ at depth at MA01 is just outside the 4.9 µmol kg^-1^ error, and 5 µmol kg^-1^ at Banana reef is also just outside error limits of 3.7 µmol kg^-1^.**

S5. Location of the LS and LN sites

Additional information on the depth profile of the carbonate mound and the location of the “time-series” stations is provided in Fig. S7.


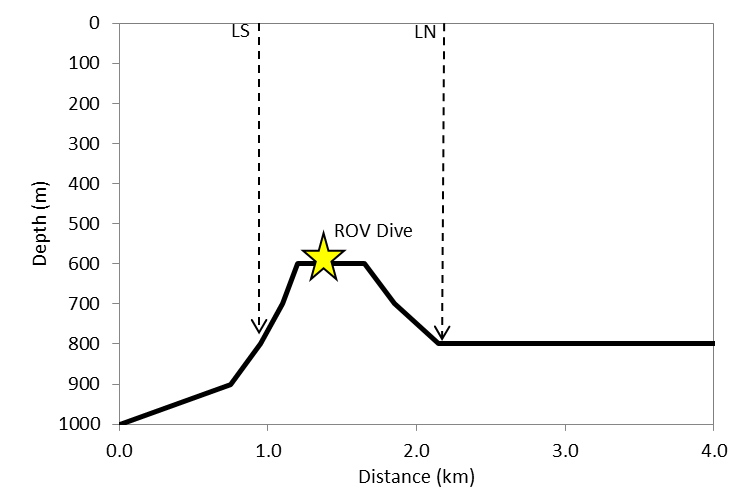


Figure S7: Depth profile of the carbonate mound investigated at Logachev, shown along a transect from north to south. Stations LS and LN are indicated by dashed arrows. The star represents the location of an ROV dive that took place on top of the carbonate mound. *Lophelia* reefs were prevalent across the mound.
